# Supplementary material for: The interactome of intact mitochondria by cross-linking mass spectrometry provides evidence for coexisting respiratory supercomplexes
Source: Mol Cell Proteomics. 2017 Dec 8;17(2):216–32. doi: 10.1074/mcp.RA117.000470 (PMC5795388; doi:10.1074/mcp.RA117.000470)
Supplement: Supplemental Data [file supp_RA117.000470_133922_0_supp_23978_fzffwf.zip › spectra_annotation/mito_spectra_annotation/212-1-8-1-9-1.pdf]

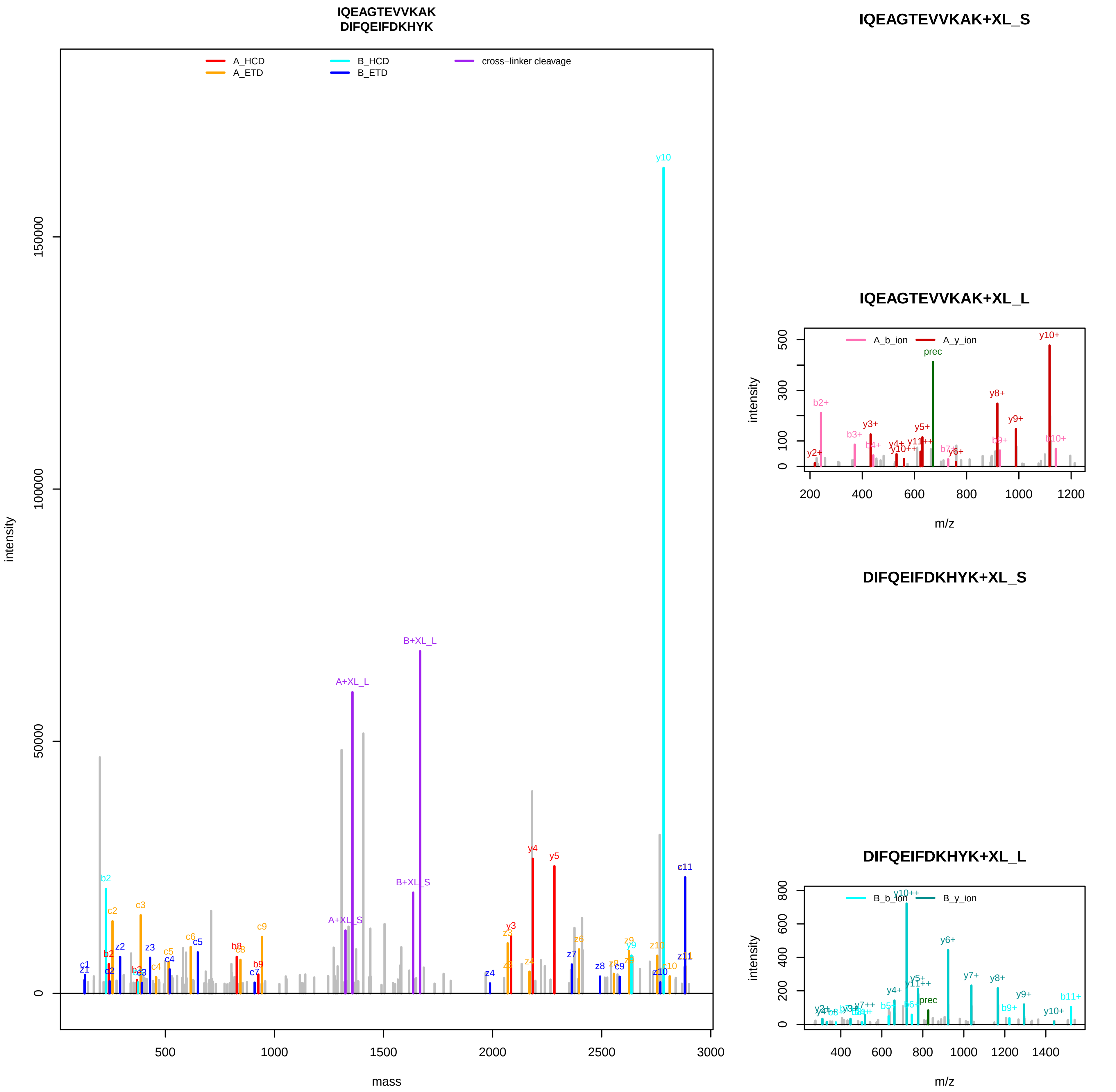
IQEAGTEVVKAK
DIFQEIFDKHYK
A_HCD
A_ETD
B_HCD
B_ETD
cross−linker cleavage
y10
B+XL_L
A+XL_L
B+XL_S
A+XL_S
y4
y5
c11
intensity
mass
0
50000
100000
150000
500
1000
1500
2000
2500
3000
IQEAGTEVVKAK+XL_S
IQEAGTEVVKAK+XL_L
A_b_ion
A_y_ion
prec
y10+
y8+
y9+
b2+
y3+
b3+
y5+
y2+
y6+
b9+
b10+
m/z
200
400
600
800
1000
1200
0
100
300
500
DIFQEIFDKHYK+XL_S
DIFQEIFDKHYK+XL_L
B_b_ion
B_y_ion
y10++
y6+
y7+
y8+
y9+
y10+
b11+
b9+
y4+
y5+
y11++
prec
0
200
400
600
800
1400
